# NHS and an SME cooperating on telehealth innovation

**Published:** 2012-06-15

**Authors:** Keith Chessell

**Affiliations:** Solcom Limited, UK

**Keywords:** SME, telehealth, innovation, portability, multi-lingual

## Abstract

**Introduction:**

Telehealth is an ideal way to lessen the burden on healthcare provision whilst empowering the patient with greater independence, assurance and control. This paper describes the joint development project between NHS South Central SHA Innovation Team and Solcom Limited, an SME specialising in bringing latest Information Technologies to healthcare.

**Aims and objectives:**

The aim of the project was to develop a low-cost Telehealth solution that makes the roll-out of Telehealth services easy and economical. The idea was to use the latest IT and communications technologies to achieve those aims. The clinician monitoring solution is internet cloud based whilst patients utilise 3G smartphones. It is also open for connection to other solutions already used by the NHS and is now connected to the Florence™ SMS based system developed by the NHS Stoke-on-Trent Simple Telehealth Program.

**Results:**

The result of the project is a Telehealth market cost and technology leader service Whzan Telehealth Service. Whzan Telehealth is:

- Budget friendly, the service starting from £1 a day
- Portable and easy to use via a smartphone or tablet PCs
- Deployed from the surgery without any home installation
- Flexible for users and healthcare professionals alike
- Disease and instrumentation agnostic
- Uniquely applicable to extended patient groups, such as dementia sufferers, expectant others and post-operative early release patients
- Secure and personalised for all users
- Multi-lingual serving ethnic minorities and user-friendly for patients with dementia, motor-skill problems or who are partially sighted

**Conclusions:**

Whzan Telehealth Service has now been used by a number of patients with long-term chronic conditions and monitored on a PCT level. A GP has deployed the service to ‘Frequent flyer’ COPD patient having 2–4 unplanned hospital admissions a week resulting in a dramatic total cessation of emergency calls. One admission costs the NHS more than 3 years of the Whzan service. It is about to be deployed to post-operative patients released early from hospital. Patients find Whzan Telehealth easy and discreet to use. They like the security offered by a Telehealth service at the same time as they appreciate the freedom provided by the portability of Whzan. The carry case for instruments is seen as practical and helping keep the monitoring discreet. Non-native English speakers in the patient group have encouraged the development of the audible and written instructions in multiple languages. A simple user interface is clear and understandable and patients are able to use the system after a very brief demonstration. Instrumentation is wireless and operation is completely automatic. Healthcare professionals appreciate the simplicity in deployment as patients can literally walk out of their appointments with the Whzan carry out pack and use the service at home. Patient management is via a web-based triage system showing everything from patient readings to equipment battery status. Clinicians can remotely change the measurement regime to suit the patient’s symptoms. Whzan also links to a third party interactive voice telephone system that can be used to provide alerts or front line patient management.

